# Pharmacological inhibition of myostatin improves skeletal muscle mass and function in a mouse model of stroke

**DOI:** 10.1038/s41598-017-13912-0

**Published:** 2017-10-25

**Authors:** Marine Maud Desgeorges, Xavier Devillard, Jérome Toutain, Josiane Castells, Didier Divoux, David Frédéric Arnould, Christopher Haqq, Myriam Bernaudin, Anne-Cécile Durieux, Omar Touzani, Damien Gilles Freyssenet

**Affiliations:** 1 Université de Lyon, Laboratoire Interuniversitaire de Biologie de la Motricité, Saint Etienne, F-42023 Lyon France; 2 Normandie Univ, Unicaen, Cea, Cnrs, Istct/Cervoxy Group, Caen, F-14000 France; 3 Atara Biotherapeutics, Inc., South San, Francisco, CA 94080 USA

## Abstract

In stroke patients, loss of skeletal muscle mass leads to prolonged weakness and less efficient rehabilitation. We previously showed that expression of myostatin, a master negative regulator of skeletal muscle mass, was strongly increased in skeletal muscle in a mouse model of stroke. We therefore tested the hypothesis that myostatin inhibition would improve recovery of skeletal muscle mass and function after cerebral ischemia. Cerebral ischemia (45 minutes) was induced by intraluminal right middle cerebral artery occlusion (MCAO). Swiss male mice were randomly assigned to Sham-operated mice (n = 10), MCAO mice receiving the vehicle (n = 15) and MCAO mice receiving an anti-myostatin PINTA745 (n = 12; subcutaneous injection of 7.5 mg.kg^−1^ PINTA745 immediately after surgery, 3, 7 and 10 days after MCAO). PINTA745 reduced body weight loss and improved body weight recovery after cerebral ischemia, as well as muscle strength and motor function. PINTA745 also increased muscle weight recovery 15 days after cerebral ischemia. Mechanistically, the better recovery of skeletal muscle mass in PINTA745-MCAO mice involved an increased expression of genes encoding myofibrillar proteins. Therefore, an anti-myostatin strategy can improve skeletal muscle recovery after cerebral ischemia and may thus represent an interesting strategy to combat skeletal muscle loss and weakness in stroke patients.

## Introduction

Stroke is the second cause of death and the leading cause of disability in industrialized countries. Every year, approximately 17 million people worldwide will have a stroke, and nearly 6 million will not survive^1^. Among stroke survivors, 50% of patients suffer from hemiparesis and 30% remain unable to walk without assistance^2^. Loss of skeletal muscle mass and function is a severe complication in stroke patients^3–5^ that promotes physical inactivity and disability, prolongs hospitalization, and limits the efficiency of rehabilitation strategies. The development of new therapeutic strategies for stroke patients obviously requires a better understanding of the pathophysiological mechanisms involved in neuronal death following cerebral ischemia, but the development of alternative therapies that could counteract/attenuate the severity and progression of muscle wasting, in combination with rehabilitation, is also needed to improve motor recovery and life quality of stroke patients.

Myostatin, also referred to as growth differentiation factor (GDF-8), belongs to the transforming growth factor-ß (TGF-ß) superfamily. Since its discovery^6,7^, myostatin has emerged as a master negative regulator of skeletal muscle growth during postnatal developmental^6,7^ and skeletal muscle mass in adulthood^8,9^. In agreement with the negative regulatory function of myostatin on skeletal muscle mass, an increase in myostatin expression is the molecular signature of multiple pathological conditions leading to a loss of skeletal muscle mass in experimental and clinical studies^10–15^, and genetic or pharmacological inhibition of myostatin has been successfully used to prevent or limit the decrease in skeletal muscle mass occurring in a number of mouse models of muscle wasting^16–21^.

All these data indicate that myostatin is a target of choice to combat skeletal muscle atrophy in numerous pathologies associated with skeletal muscle deconditioning. However, the question whether myostatin inhibition could be used as therapeutical intervention in stroke remains unanswered. Therefore, we hypothesized that inhibition of myostatin would improve skeletal muscle mass and function after cerebral ischemia. In support of this assumption, myostatin expression has been shown to be elevated in skeletal muscles of the paretic limb compared to the non-paretic limb of stroke patients^22^. More recently, we also showed that myostatin expression was markedly increased in skeletal muscle 3 days after transient cerebral ischemia in mice^23^. In the present study, we therefore determined the potential therapeutic impact of myostatin inhibition in a mouse model of stroke by administration of PINTA745, a genetically engineered myostatin-neutralizing peptide fused to a Fc fragment.

## Results

### PINTA745 reduces body weight loss and improves body weight recovery after cerebral ischemia

**Figure 1:**
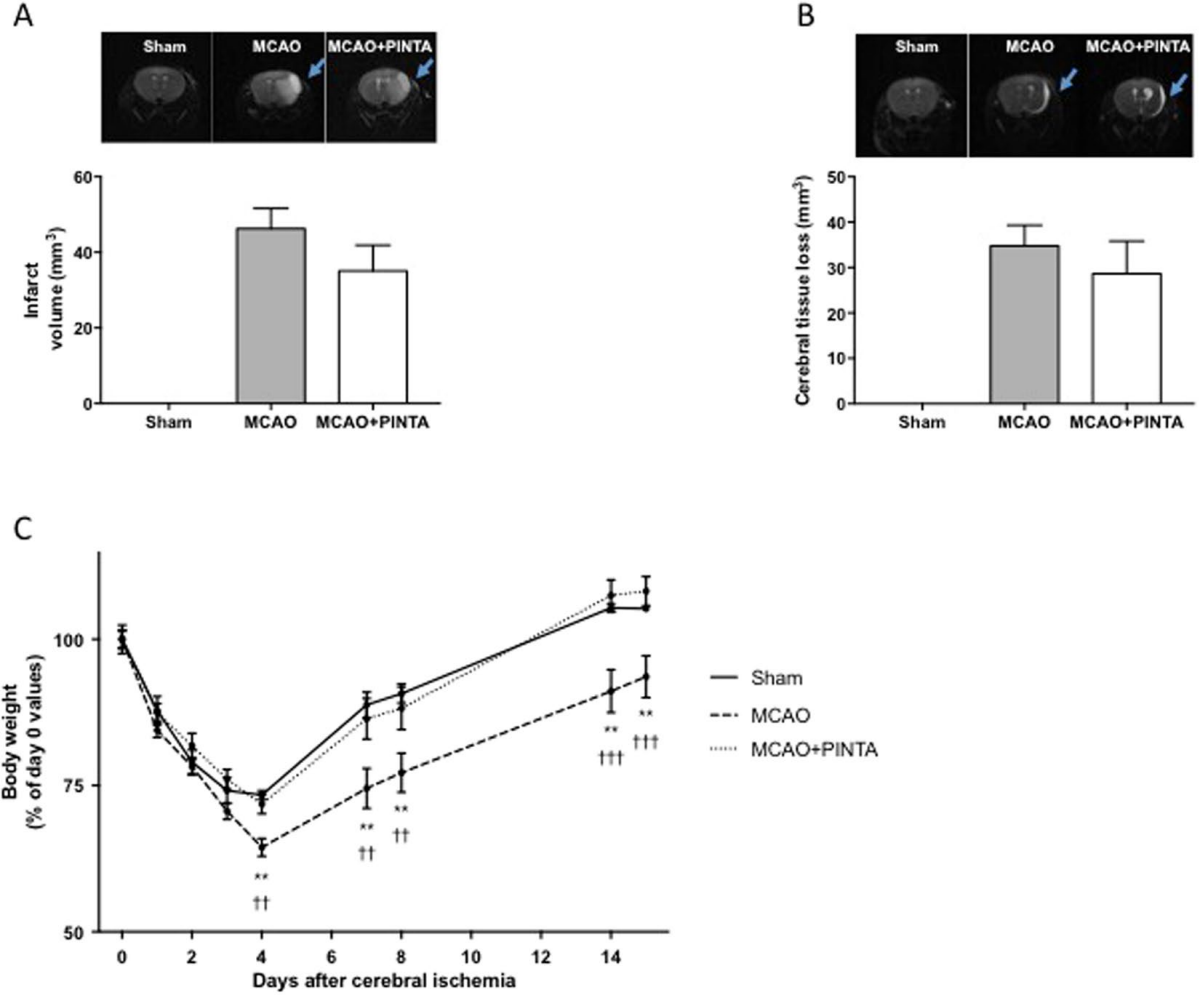
PINTA745 improves recovery of body weight following middle cerebral artery occlusion (MCAO). (**A**) Infarction in the right hemisphere (upper panel) and quantification of cerebral infarct volume (lower panel) 2 days after MCAO. (**B**) Infarction in the right hemisphere 14 days after MCAO (upper panel) and quantification of cerebral tissue loss. Cerebral tissue loss was expressed as the difference between contralateral and ipsilateral hemispheric volumes at day 14 after MCAO. Data are means ± SEM (*n* = 8–14/group). (**C**) Kinetic of body weight loss and recovery after cerebral ischemia. Data are means ± SEM (n = 7–12/group). Error bars not shown are within symbol size. **P < 0.01: vehicle-MCAO mice (MCAO) significantly different from Sham mice at the corresponding time point. ^**††**^P < 0.01 and ^**†††**^P < 0.001: MCAO mice significantly different from PINTA745-MCAO mice (MCAO + PINTA) at the corresponding time point.

MCAO induced a brain lesion that affected the lateral striatum and parietal cortex (Fig. 1A). The whole brain lesion averaged 46.3 ± 5.3 mm^3^ 2 days after cerebral ischemia in vehicle-MCAO mice. Although the whole brain lesion was slightly lower in PINTA745-MCAO mice, the difference was not significant. At day 14, no difference in the volume of brain lesions was observed between the two groups (Fig. 1B).

All groups of mice lost weight after surgery, included sham-operated mice (Fig. 1C)^23^. Vehicle-MCAO mice continued to lose weight 4 days after cerebral ischemia, whereas MCAO mice treated with PINTA745 had already ceased to lose weight (P < 0.01). Furthermore, PINTA745-MCAO mice regained baseline body weight values by day 14, whereas body weight of vehicle-MCAO mice had not returned to control values at this time (P < 0.001). The kinetics of body weight loss and recovery were similar in Sham mice and PINTA745-MCAO mice.

### PINTA745 increases muscle weight recovery after cerebral ischemia

**Figure 2:**
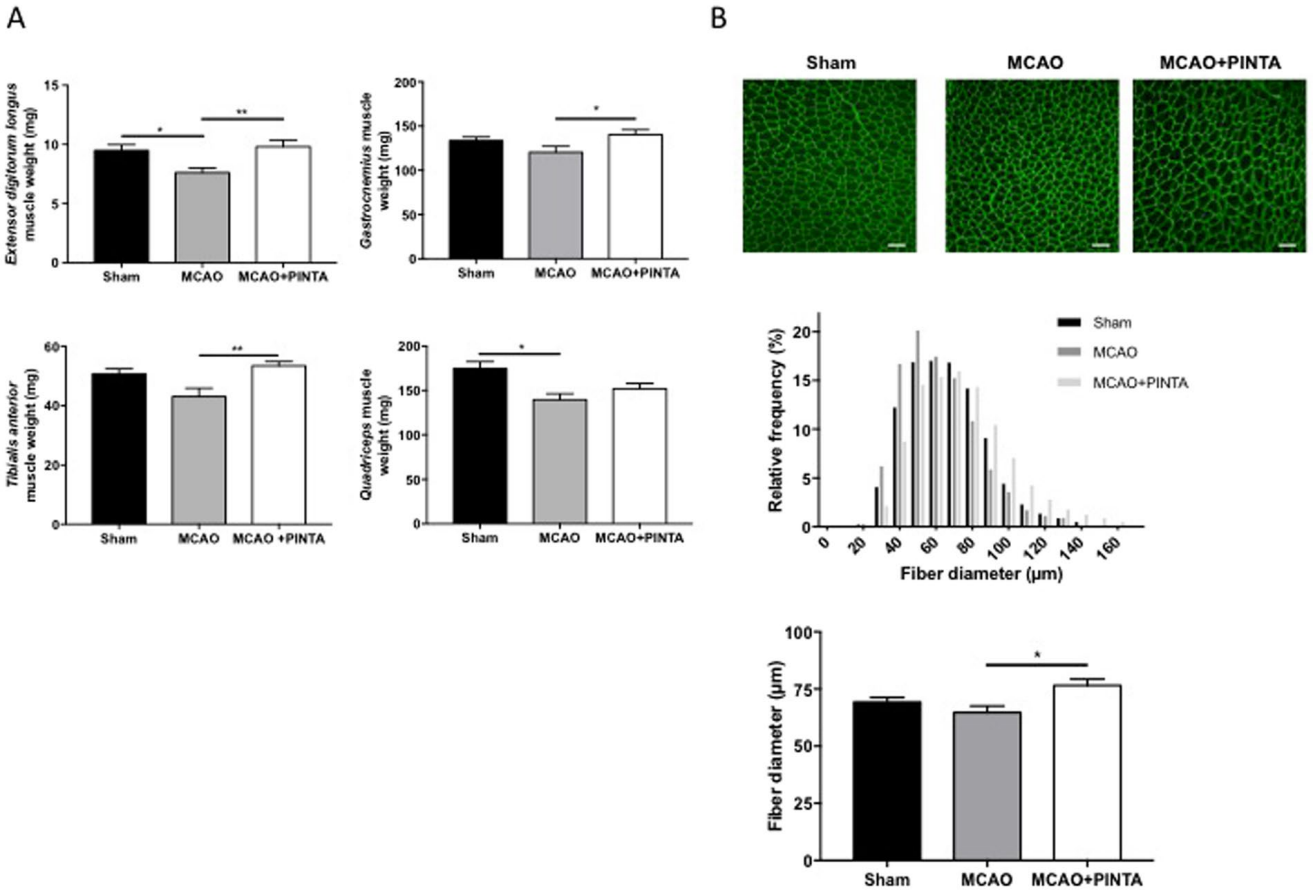
PINTA745 reduces skeletal muscle weight loss 15 days after middle cerebral artery occlusion (MCAO). (**A**) Weight of *extensor digitorum* longus, *gastrocnemius*, *tibialis anterior* and *quadriceps* muscles in Sham mice, vehicle-MCAO mice (MCAO) and PINTA745-MCAO mice (MCAO + PINTA). (**B**) Representative transverse sections of *tibialis anterior* muscle immunostained with anti-laminin antibody in Sham mice, MCAO mice and MCAO + PINTA mice. Images were acquired using a Leica TCS-SP2 confocal scanning laser inverted microscope (Leica-Microsystem, Heidelberg, Germany). Relative frequency distribution of fiber diameter and quantification of muscle fiber diameter in Sham, MCAO and MCAO + PINTA mice. Scale bar equals 100 µm. Data are means ± SEM (n = 5/group). *P < 0.05 and **P < 0.01.

PINTA745 increased muscle weight 15 days after cerebral ischemia. This beneficial effect of myostatin blockade on skeletal muscle was observed in *extensor digitorum longus*, *gastrocnemius* and *tibialis anterior* muscles, whose weights were significantly higher in PINTA745-MCAO mice compared to vehicle-MCAO mice (Fig. 2A). This beneficial effect of myostatin blockade was also observed in *quadriceps* muscle whose weight remained unchanged between Sham mice and PINTA745-MCAO mice, whereas it was significantly lower in vehicle-MCAO mice compared to Sham mice (P < 0.05) (Fig. 2A). In agreement with these data, our immunohistological analyse indicated that the fiber diameter of *tibialis anterior* muscle was significantly higher in MCAO mice treated with PINTA745 compared to vehicle-MCAO mice (Fig. 2B) (P < 0.05). Together with our previous published observation that MCAO induced a marked atrophy of skeletal muscle 3 days after cerebral ischemia^23^, our data clearly indicate that PINTA745 increases the recovery of skeletal muscle weight in response to cerebral ischemia.

### IGF-1-Akt-mTOR pathway, autophagy-lysosome and ubiquitin-proteasome systems 15 days after cerebral ischemia

**Figure 3:**
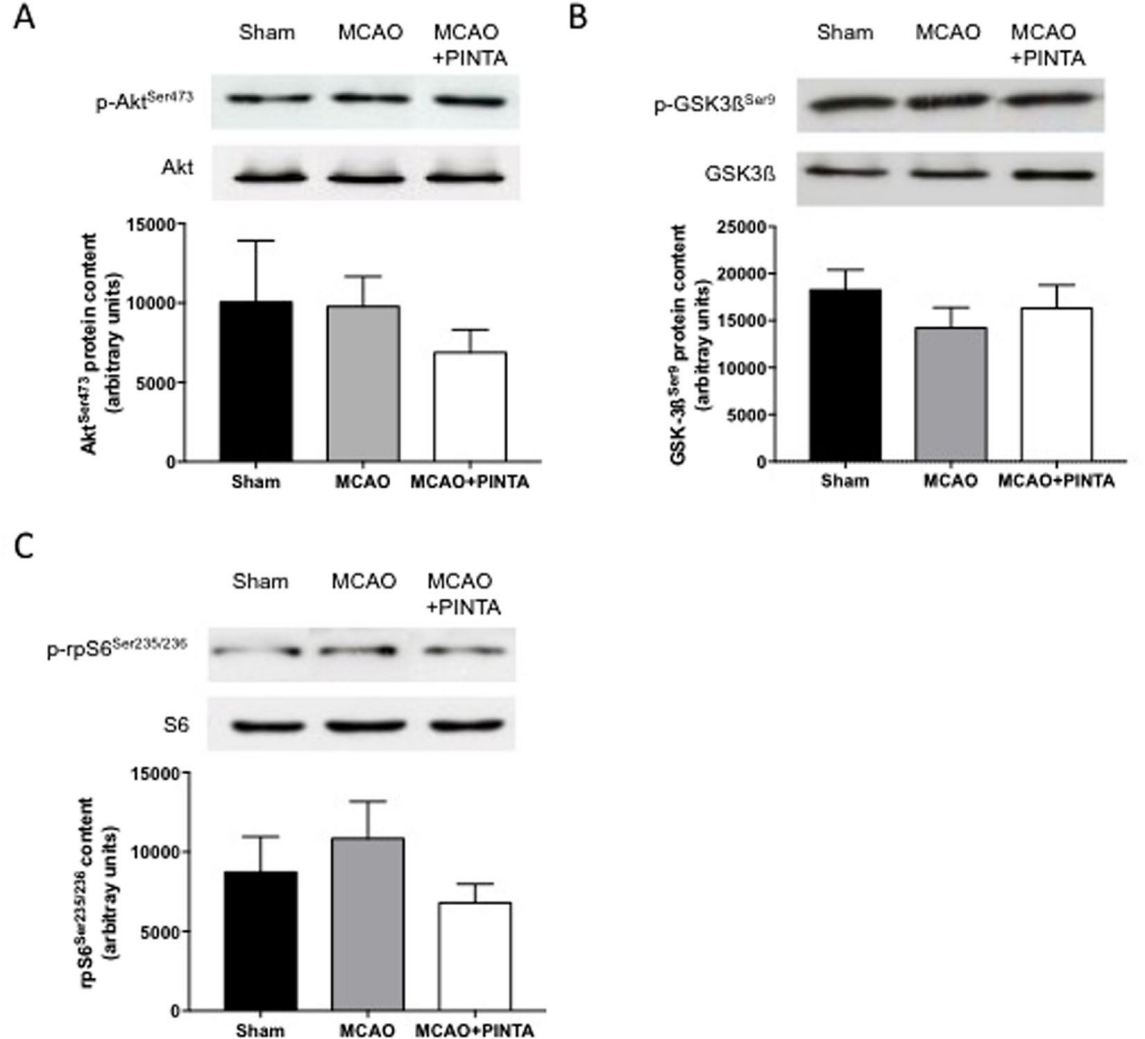
IGF-1-Akt-mTOR pathway. Representative immunoblots (upper panel) and quantification (lower panel) of phosphorylated Akt^Ser473^ (**A**), GSK3ß^Ser9^ (**B**) and rpS6^Ser 235/236^ (**C**) protein content in *quadriceps* muscle of Sham mice, vehicle-MCAO mice (MCAO) and PINTA745-MCAO mice (MCAO + PINTA) 15 days after middle cerebral artery occlusion (MCAO). Representative immunoblots of Akt, GSK3ß and rpS6 total protein level (upper panel) indicate that total protein level remained unchanged. Data are means ± SEM (n = 6–9/group).

Skeletal muscle homeostasis mainly results from a dynamic equilibrium between protein synthesis and protein degradation. The IGF-1-Akt-mammalian target of rapamycin (mTOR) pathway is a positive regulator of protein translation initiation^24^, whose activation prevents muscle atrophy *in vivo* ^24,25^. No significant difference was reported in the phosphorylation level of Akt^Ser473^ (Fig. 3A), GSK-3ß^Ser9^ (Fig. 3B) and rpS6^Ser236/325^ (Fig. 3C) between Sham mice, vehicle-MCAO mice and PINTA745-MCAO mice 15 days after MCAO. Protein level of corresponding total forms remained unchanged.

**Figure 4:**
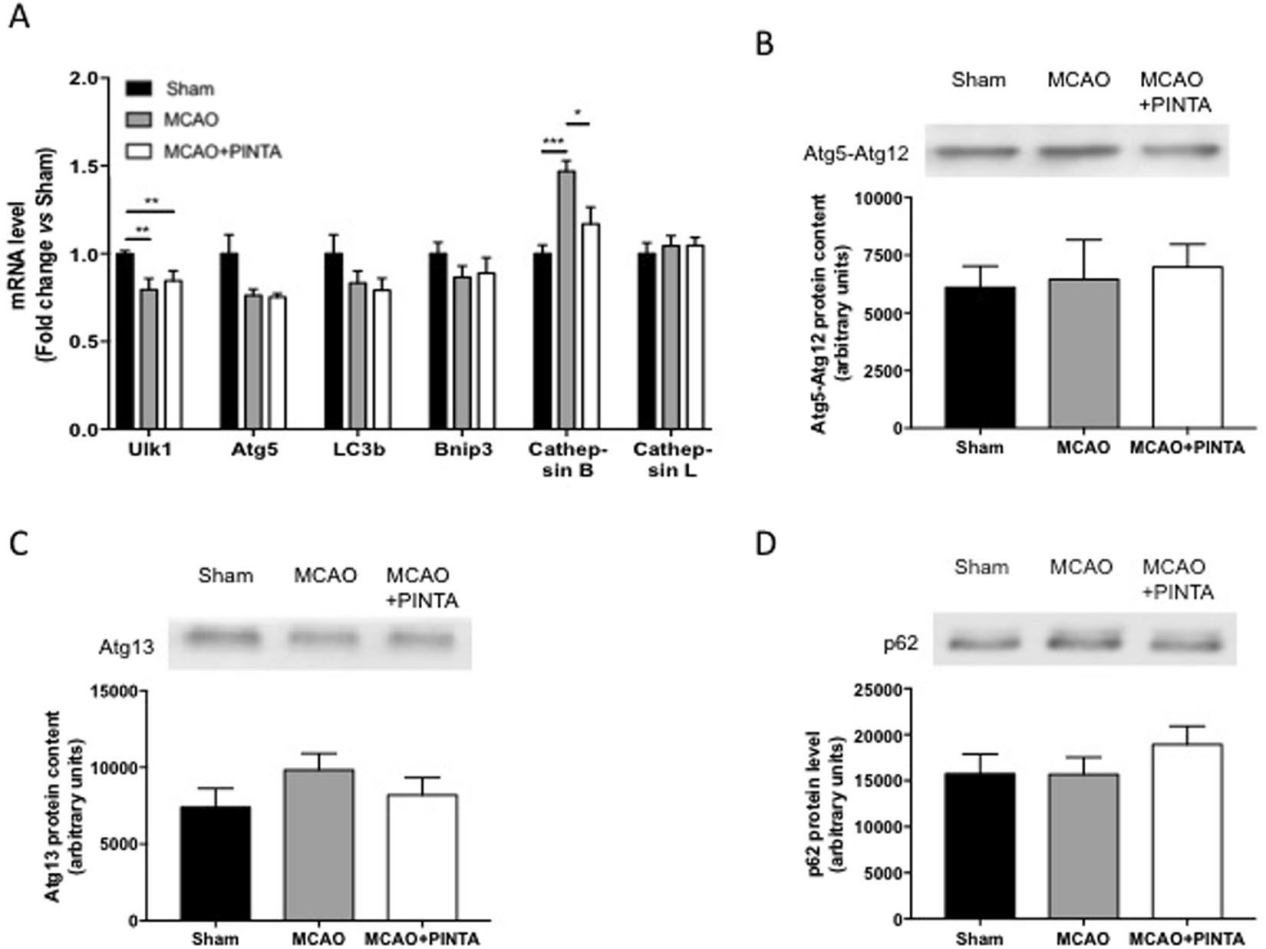
Regulation of autophagy-lysosome system. (**A**) Transcript level of Atg5, Ulk1, LC3b, Bnip3, cathepsin B and cathepsin L in Sham mice, vehicle-MCAO mice (MCAO) and PINTA745-MCAO mice (MCAO + PINTA) 15 days after middle cerebral artery occlusion (MCAO). Representative immunoblots (upper panel) and quantification (lower panel) of Atg5-Atg12 (**B**), Atg13 (**C**) and p62 (**D**) protein content in quadriceps muscle of Sham, MCAO and MCAO + PINTA mice. Data are means ± SEM (n = 6–9/group). **P < 0.01.

We next monitored the expression of autophagy-related genes involved in autophagy-lysosome proteolysis (Fig. 4). Although the transcript level of Atg5 was significantly decreased in both vehicle-MCAO mice and PINTA745-MCAO mice compared to Sham mice, Ulk1, LC3b and Bnip3 transcript level, as well as Atg5-Atg12 protein complex, Atg13 and p62 protein content remained unchanged. The transcript level of cathepsin B, a lysosomal hydrolase, was significantly increased in vehicle-MCAO mice when compared to both Sham mice and PINTA745-MCAO mice, whereas that of cathepsin L remained unchanged.

**Figure 5:**
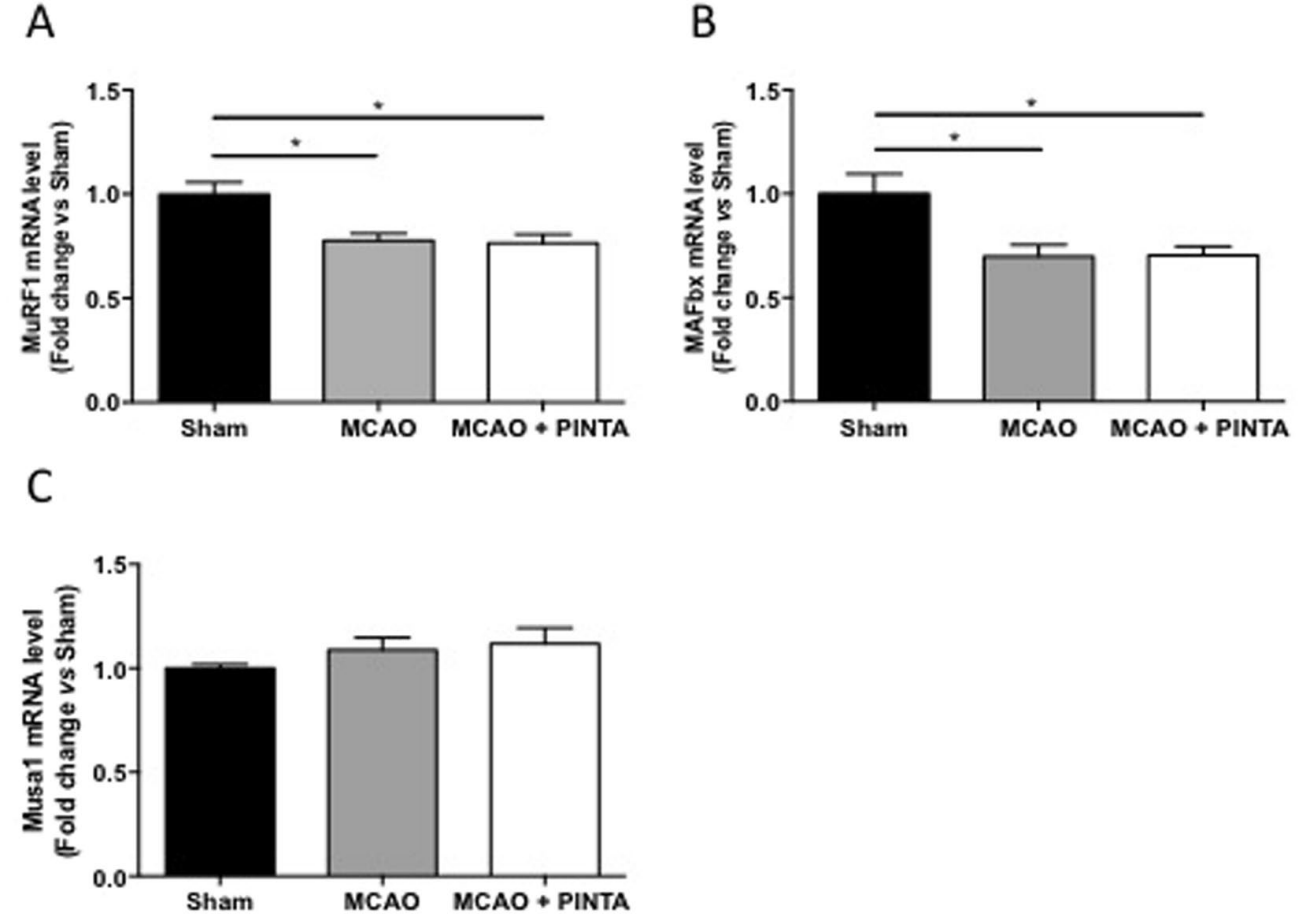
Transcripts levels of E3-ubiquitin ligases after cerebral artery occlusion (**MCAO)**. (**A**) MuRF-1 mRNA level in Sham mice, vehicle-MCAO mice (MCAO) and PINTA745-MCAO mice (MCAO + PINTA). (**B**) MAFbx mRNA level. (**C**) Musa1 mRNA level. Data are means ± SEM (n = 6–9/group). *P < 0.05.

The mRNA levels of the E3-ubiquitin ligases, muscle RING finger-1 (MuRF-1), muscle atrophy F-box (MAFbx) involved in ubiquitin-proteasome-dependent proteolysis^24,26^ were significantly decreased 15 days after cerebral ischemia in both vehicle-MCAO mice and PINTA745-MCAO mice compared to Sham mice (Fig. 5A,B, P < 0.05). By contrast, mRNA level of Musa1, another E3-ubiquitin ligase^27^, remained unchanged between groups (Fig. 5C).

### PINTA745 increases expression of myosin heavy chain isoforms after cerebral ischemia

**Figure 6:**
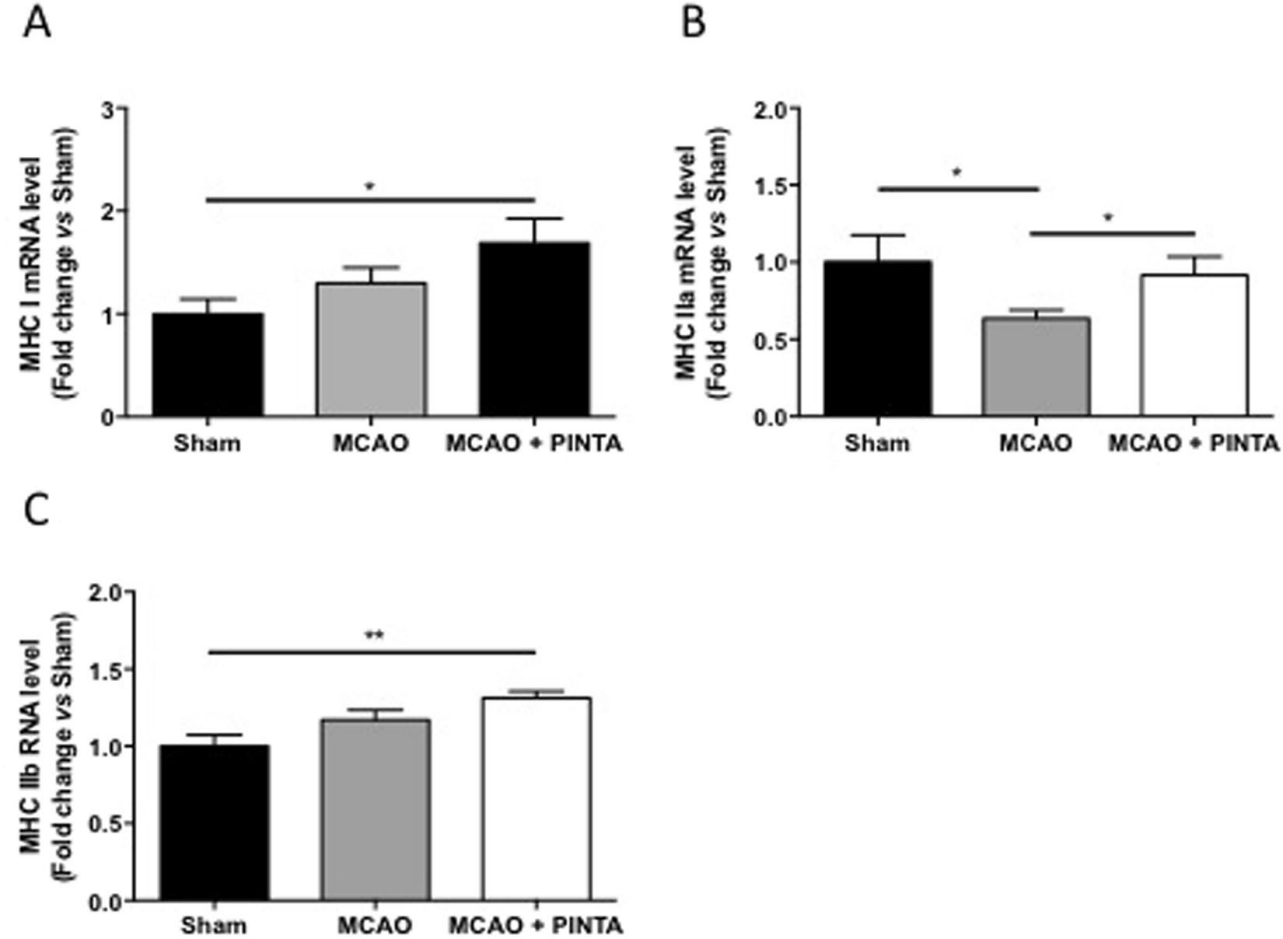
Transcript level of myosin heavy chain isoforms. Transcipt level of MHC I (**A**), MHC IIa (**B**) and MHC IIb (**C**) in *quadriceps* muscle of Sham mice, vehicle-MCAO mice (MCAO) and PINTA745-MCAO mice (MCAO + PINTA) 15 days after middle cerebral artery occlusion (MCAO). Data are means ± SEM (n = 6–9/group). *P < 0.05 and **P < 0.01.

Myostatin has been shown to reduce the expression of muscle-specific genes^9^, suggesting that a higher expression of muscle-specific gene by PINTA745 would contribute to increase the recovery of skeletal muscle mass after cerebral ischemia. We therefore investigated the transcript level of myosin heavy chain (MHC) isoforms I, IIa, and IIb, three prototypical specific-muscle genes. Expression of MHC isoforms showed an expression pattern that was improved by myostatin inhibition. Transcript level of MHC-I and MHC-IIb isoforms was significantly higher in MCAO mice compared to Sham mice, whereas no difference was observed between Sham and vehicle-MCAO mice (Fig. 6A and C). Similarly, expression of MHC-IIa was higher in Sham mice and PINTA745-MCAO mice compared to vehicle-MCAO mice (Fig. 6B).

### PINTA745 improves muscle function after cerebral ischemia

**Figure 7:**
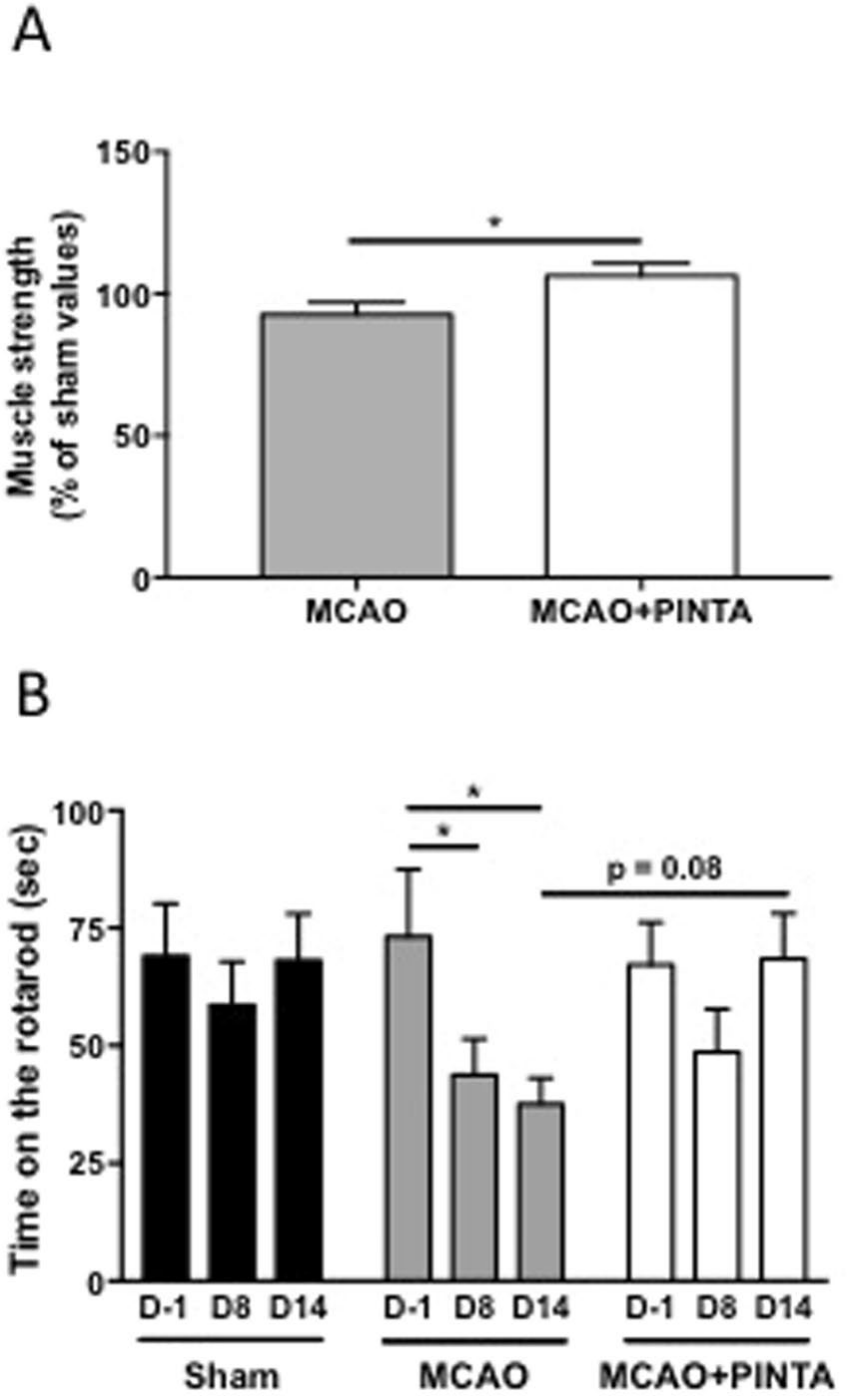
PINTA745 improves recovery of skeletal muscle function after middle cerebral artery occlusion (MCAO). (**A**) Absolute muscle strength of vehicle-MCAO mice (MCAO) and PINTA745-MCAO mice (MCAO + PINTA) 15 days after MCAO. (**B**) Performance on the Rotarod of Sham, MCAO and MCAO + PINTA mice. D-1: One day before MCAO; D8: 8 days after MCAO; D14: 14 days after MCAO. Data are means ± SEM (n = 7–11/group). *P < 0.05.

Finally, we also determined whether the better recovery in muscle mass was also associated with beneficial functional adaptations. Muscle force determined by a grip test was significantly higher in PINTA745-MCAO mice 14 days after cerebral ischemia compared to vehicle-MCAO mice (Fig. 7A, P < 0.05). The rotarod performance test provides an objective measurement of motor skills (balance, coordination, motor-planning) and is therefore particularly relevant to determine the recovery of motor function after cerebral ischemia. The time spent on the rotarod was significantly decreased in vehicle-MCAO mice 8 (−56%) and 14 (−62%) days after cerebral ischemia (Fig. 7B). By contrast, rotarod performance, which was not significantly decreased at Day 8 (−51%), returned to control values 14 days after cerebral ischemia in PINTA745-MCAO mice. Overall, these data clearly indicate that PINTA745 improved the recovery of muscle and motor functions after cerebral ischemia.

## Discussion

In the present study, we report the effect of an anti-myostatin strategy on the regulation of skeletal muscle mass and function in a mouse model of stroke. Our results demonstrate that subcutaneous injection of PINTA745, an anti-myostatin PINTA745, improves body weight recovery and increases skeletal muscle mass 15 days after transient cerebral ischemia. We also demonstrate that these effects were associated with an increase in skeletal muscle strength and an improvement in the recovery of motor function. Thus, our findings suggest that an anti-myostatin strategy represents a potential therapeutical approach to limit the deleterious effects of stroke on skeletal muscle mass and function.

Based on the analysis of body weight after cerebral ischemia, 2 different phases can be distinguished, a catabolic phase (Day 0 to Day 4 after cerebral ischemia) associated with a body weight loss, and an anabolic phase (Day 5 to Day 15 after cerebral ischemia) associated with a progressive recovery of body weight. Clearly, PINTA745 limited the extent of body weight loss and greatly improved the recovery of body weight following cerebral ischemia. Skeletal muscle weights were also significantly higher in MCAO mice receiving PINTA745 compared to MCAO mice receiving the vehicle 15 days after cerebral ischemia. Together with our previous published observation that skeletal muscle mass was decreased 3 days after cerebral ischemia^23^, these data indicate PINTA745 limited the extent of muscle mass loss and/or improved the recovery of skeletal muscle mass. Overall, these data illustrate the efficiency of an anti-myostatin strategy in this mouse model of stroke.

Myostatin is a master negative regulator of skeletal muscle growth during development. Myostatin knockout mice display a 2–3-fold increase in muscle mass that result from a combination of both hypertrophy and hyperplasia^6^. Similarly, naturally occurring mutations in myostatin results in hypertrophic phenotype in several species^7,28–31^ including human^32^. Beyond its developmental effects, myostatin also regulates skeletal muscle mass homeostasis in adulthood. Myostatin inhibition increases muscle mass in adult mouse^33,34^, whereas myostatin overexpression induces atrophy of skeletal muscle in adult rat^8,9^. Furthermore, previous studies showed the efficiency of genetic and pharmacological anti-myostatin strategies in several mouse models of skeletal muscle wasting^16–21,35^. Altogether, these data agree with our observation of increased muscle mass in MCAO mice treated with PINTA745.

Mechanistically, the better recovery of skeletal muscle mass in MCAO mice receiving PINTA745 cannot be explained by a specific regulation of IGF-1-Akt-mTOR pathway and autophagy-lysosome system 15 days after cerebral ischemia. However, we cannot exclude that a regulation of these pathways could occur earlier during the catabolic phase and/or the recovery phase. By contrast, the transcript levels of MuRF-1 and MAFbx were all decreased in vehicle-MCAO mice and PINTA745-MCAO mice, strongly suggesting that a reduction of ubiquitin-proteasome-dependent proteolysis was probably involved in the recovery process that occurs in both vehicle- and PINTA-745-MCAO mice. This also indicates that the better recovery of skeletal muscle mass with PINTA745 cannot be explained by a specific down-regulation in the expression level of MuRF-1 and MAFbx in PINTA745-MCAO mice at least at this time point. An analysis of this transcriptional response earlier during cerebral ischemia would provide a definite answer about the role of ubiquitin-proteasome system in the better recovery of skeletal muscle mass with PINTA745.

Myofibrillar proteins represent more than 80% of muscle fiber volume^36^. Any loss of myofibrillar proteins will therefore greatly impact skeletal muscle mass. The greater transcript levels of MHC isoforms (MHC I, MHC IIa and MHC IIb) in PINTA745-MCAO mice compared to vehicle-MCAO mice suggests that PINTA745 could improve skeletal muscle mass after cerebral ischemia by triggering the expression of myofibrillar genes, which could then sustain a better accretion of myofibrillar proteins. Our findings are also in agreement with previous data showing that myostatin overexpression decreased muscle-specific gene expression^9^.

As expected, the increase in skeletal muscle mass in PINTA745-MCAO mice was also associated with an increase in skeletal muscle strength. Although an increase in muscle strength is not systematically observed in response to the pharmacological inhibition of myostatin^37^, a number of studies have clearly described an increase in muscle strength following an anti-myostatin treatement in experimental model of muscle wasting, including *mdx* Duchenne muscular dystrophy mice^21,38^, mouse model of cancer cachexia^11,17^, or mice aging^20^. Importantly, our study shows for the first time that severe complications of cerebral ischemia in mice, including losses of muscle mass and strength, can be efficiently restored after 2 weeks of treatment with PINTA745.

PINTA745 also improved motor the recovery of motor skills 15 days after cerebral ischemia. Whereas rotarod performance declined over the time course of the experiment in vehicle-MCAO mice and remained significantly below control values at Day 14, rotarod performance was not significantly affected higher in PINTA745-MCAO mice. Although the increased skeletal muscle mass in PINTA745-MCAO mice may contribute to improve rotarod performance, increasing skeletal muscle mass by PINTA745 may also contribute to some unexpected neurogenic effects. In support of this assumption, it has been shown that rotarod performance was correlated to motor cortex plasticity in mice after rotarod training^39^. Furthermore, delivery of recombinant follistatin, a natural antagonist of myostatin, in a mouse model of spinal muscular atrophy, has been shown to increase the number and size of ventral horn cells together with the gross motor function of mice^40^. Therefore, myostatin inhibition by PINTA745 either directly, but also indirectly via its muscle-enhancing activity may trigger a neurogenic effect that contributes to enhance motor coordination as assessed by rotarod performance. Further experiments will be necessary to explore this hypothesis.

In summary, our data provide evidence that a short-term treatment with a myostatin inhibitor (PINTA745) can increase body weight recovery and skeletal muscle mass in a mouse model of stroke. This treatment also increases skeletal muscle strength and recovery of motor function after cerebral ischemia. Altogether, these data point to the potential benefit of this therapeutic approach to combat the loss of muscle mass and muscle function that occurs in human stroke patients.

## Methods

### Animals and induction of cerebral ischemia

The investigations were performed under the current European directive (2010/63/UE) and approved by the Regional Ethic Committee (CENOMEXA). Animals were housed in the Central Animal Care Facility of Caen-Normandie University (France). Under isoflurane anesthesia (1.5% in N_2_O/O_2_), transient focal cerebral ischemia (45 minutes) was induced by intraluminal right middle cerebral artery occlusion (MCAO)^41^ in 13-week-old Swiss male mice (n = 27). A nylon thread (0.08 mm diameter) with a terminal cylinder of melting glue (3 mm length, 0.24 mm diameter) was inserted into the lumen of the external carotid artery, and then gently advanced into the internal carotid artery up to the origin of the middle cerebral artery. Forty-five minutes later, the animals were re-anesthetized and the nylon thread was gently removed allowing the reperfusion of the ischemic brain area. In Sham animals (n = 10), the nylon thread was introduced up to the origin of the middle cerebral artery and then immediately removed. Sham-operated animals were also anesthetized 45 minutes after surgery. After suture of the skin, animals were rapidly allowed to recover from anesthesia in their cage. Shortly after surgery and every day following the surgery (during 7 days), mice received 1 ml of physiological saline subcutaneously.

### Injection of PINTA745

PINTA745, the anti-myostatin PINTA745, was provided by Atara Biotherapeutics (South San Francisco, CA, USA). PINTA745 is a protein construct made of a synthetic peptide fused to an antibody that binds to and specifically blocks myostatin. PINTA745 was injected subcutaneously at 7.5 mg.kg^−1^ (in 0.9% sterile-endotoxin free NaCl) immediately after surgery, 3, 7 and 10 days after MCAO (MCAO + PINTA, *n* = 12). The other paired mice were injected with an equal volume of vehicle (MCAO, *n* = 15). Sham mice also received the vehicle (Sham, *n* = 10).

### Magnetic resonance imaging analysis

T2-weighted MR images were acquired (TE/TR = 46 ms/5000 ms, number of excitation 6, field of view 20 mm 2, matrix 256 × 192, 15 slices, thickness 0.75 mm) on anesthesized mice (1.5% in N_2_O/O_2_) 2 days and 14 days following the induction of stroke using a 7-T Pharmascan (Bruker, CYCERON Biomedical Imaging platform). Images were then used to generate T2 maps. The delineated lesion areas were summed and multiplied by the slice thickness to determine the infarct volume at day 2 or tissue loss at day 14.

### Rotarod and grip test

Muscle strength was assessed 14 days after surgery by a grip test^23^. Peak force of both forelimbs and hindlimbs was recorded on 3 consecutive trials and the best of 3 consecutive trials was used for analysis.

Motor coordination was analyzed through the use of the Rotarod test. After an acclimatization period, each mouse was tested on the rotarod at a constant speed of 4 rpm the day before surgery, 8 and 14 days after surgery. Time before falling was recorded on 3 consecutive trials. The mean of the 3 consecutive trials was used for analysis.

### Tissue removal

Fifteen days after surgery, animals were anesthesized (*i*.*p*. injection of 90 mg.kg^−1^ ketamine and 10 mg.kg^−1^ xylazine). *Extensor digitorum longus*, *gastrocnemius*, and *quadriceps* muscles were removed, weighted and stored at −80 °C. *Tibialis anterior* muscle was weighted, mounted in embedding medium and frozen in isopentane for subsequent immunochemical analyses of muscle fiber morphometry.

### Immunochemistry

*Tibialis anterior* muscles were cut (12 μm) in a cryostat (Leica CM 1950). Transverse sections were fixed in 4% paraformaldehyde and incubated with antilaminin (1:200; Sigma-Aldrich, Saint-Quentin Fallavier, France). Fluorescent muscle fibers were visualized with a MVX10 microscope (Olympus) connected to a XM10 monochrome camera (Olympus). Muscle fiber ferret diameter was determined on 1,685 ± 105.6 fibers per muscle by using Image J software (https://imagej.nih.gov/ij/).

### RNA isolation, cDNA synthesis and real-time polymerase chain reaction

Total RNA extraction, synthesis of cDNA, and real-time quantitative polymerase chain reaction was performed as previously described on *quadriceps* muscle^42^. Briefly, total RNA was extracted from 15–20 mg of *quadriceps* muscle using the QIAzol Lysis Reagent (Qiagen, Courtaboeuf, France) and NucleoSpin RNA (Macherey-Nagel, Hoerdt, France). cDNA was generated from 200 ng of RNA using Reverse transcriptase Core kit (Eurogentec, Angers, France). The selected forward and reverse primer sequences are listed in Table 1. Real time PCR was performed in a 20 µl final volume and optimized concentrations for each primer using the SsoFast EvaGreen Super mix (Bio-Rad) and a CFX96 Real Time PCR Detection System, C1000 Thermal Cycler (Bio-Rad). Peptidylprolyl isomerase A and β-actin were used as reference genes^43^.

**Table 1:** Oligonucleotide primers used for PCR analysis.

Gene	Primer sequence 5′-3′	GenBank^®^ accession no.
β-actin	Fwd: AGCAAGCAGGAGTACGATGAG	NM_007393.3
	Rev: AACGCAGCTCAGTAACAGTC	
Atg5	Fwd: TGAAAGAGTGTGTCCTCCTC	NM_053069.5
	Rev: GCCTCCACTGAACTTGACTG	
Bnip3	Fwd: AGA TTG GAT ATG GGA TTG GTC AAG	NM_009760.4
	Rev: CCC TTT CTT CAT AAC GCT TGT G	
Cathepsin B	Fwd: GAA GAA GCT GTG TGG CAC TG	NM_007798.3
	Rev: GTT CGG TCA GAA ATG GCT TC	
Cathepsin L	Fwd: GTG GAC TGT TCT CAC GCT CAA G	NM_009984.3
	Rev: TCC GTC CTT CGC TTC ATA GG	
LC3b	Fwd: CACTGCTCTGTCTTGTGTAGGTTG	NM_026160.4
	Rev: TCGTTGTGCCTTTATTAGTGCATC	
MAFbx/atrogin-1 (Fbxo32)	Fwd: GTTTTCAGCAGGCCAAGAAG	NM_026346.3
	Rev: TTGCCAGAGAACACGCTATG	
MHC I	Fwd: AGTCCCAGGTCAACAAGCTG	NM_080728.2
	Rev: TTCCACCTAAAGGGCTGTTG	
MHC IIa	Fwd: AGTCCCAGGTCAACAAGCTG	NM_001039545.2
	Rev: GCATGACCAAAGGTTTCACA	
MHC IIb	Fwd: AGTCCCAGGTCAACAAGCTG	NM_010855.3
	Rev: TTTCTCCTGTCACCTCTCAACA	
MuRF1 (Trim63)	Fwd: ACCTGCTGGTGGAAAACATC	NM_001039048.2
	Rev: AGGAGCAAGTAGGCACCTCA	
Musa1	Fwd: TCGTGGAATGGTAATCTTGC	NM_001168297.1
	Rev: CCTCCCGTTTCTCTATCACG	
Ppia	Fwd: AGCATACAGGTCCTGGCATC	NM_008907.1
	Rev: TTCACCTTCCCAAAGACCAC	
Ulk1	Fwd: TTCCTGTCAGTCTGGCTCCT	NM_009469.3
	Rev: TGAACAGAGCCGTGACAAAG	

Atg5: autophagy related 5; Bnip3: BCL2 interacting protein3; Fbxo30/Musa1: F-box protein 30/muscle ubiquitin ligase of the SCF complex in atrophy-1; MAFbx/atrogin-1: F-box protein 32; Map1lc3b/LC3b, microtubule-associated protein 1 light chain 3 beta. MHC I: type I myosin heavy chain; MHC IIa: type IIa myosin heavy chain; MHC IIb: type IIb myosin heavy chain; MuRF1 (Trim63): tripartite motif-containing 63; Ulk1: unc-51 like kinase 1. Ppia: peptidylprolyl isomerase A.

### Protein extraction and immunoblotting

*Quadriceps* muscles were homogenized (1:20 dilution wt/vol) in ice-cold buffer (50 mmol·l^−1^ Tris HCl pH = 7.4, 100 mmol·l^−1^ NaCl, 2 mmol·l^−1^ EDTA, 2 mmol·l^−1^ EGTA, 50 mmol·l^−1^ β-glycerophosphate, 50 mmol·l^−1^ sodium fluoride, 1% Triton X-100, 1 mmol·l^−1^ sodium orthovanadate, and 120 nmol·l^−1^ okadaic acid, all reagents from Sigma-Aldrich). Homogenates were centrifuged at 12,000 g for 20 min at 4 °C, and the resulting supernatants analyzed for protein content (Bio-Rad DC Protein Assay) using bovine serum albumin as standard. Equal amount of protein (50 µg) were resolved on 12.5% SDS-polyacrylamide gels. Proteins were transferred to 0.45 µm nitrocellulose membranes. After Ponceau S staining, membranes were blocked 1 h at room temperature with Tris-buffered saline (TBS) containing 5% non-fat dried milk, and then incubated overnight with antibodies directed against Akt^Ser473^ (1:500, Cell Signaling Technology, Danvers USA, CS9271), Akt (1:500, Cell Signaling Technology, CS9272), Atg5-Atg12 conjugate (1:800, Sigma-Aldrich, A0856), Atg13 (1:800, Sigma-Aldrich, SAB4200100), GSK-3ß^Ser9^ (1:500, Cell Signaling Technology, CS9336), GSK-3ß (1:800, Cell Signaling Technology, CS9315), p62 (1:800, Sigma-Aldrich, P0067), rpS6^Ser235/236^ (1:1000, Cell Signaling Technology, CS4856), rpS6 (1:1000, Cell Signaling Technology, CS2217), and α-tubulin (1:1500, Sigma-Aldrich, T5168). Membranes were then washed 3 times in TBS and incubated for 1 h at room temperature with a goat anti-rabbit or a rabbit anti-mouse horseradish peroxidase-conjugated secondary antibody (Dako, Trappes, France). After 3 × 5 min washes in TBS-Tween, immunocomplexes were visualized using an enhanced chemiluminescence detection method (GE Healthcare, Orsay, France). The films were scanned and quantified using ImageJ analysis software (http://rsb.info.nih.gov/ij/). α-tubulin immunoblots were used to check for equal protein loading between samples.

### Statistical analysis

Statistical analyses were performed using GraphPad PRISM 5.0 (La Jolla, CA, USA). Data were analyzed for normal distribution using Shapiro-Wilk test. Two-way ANOVA followed by Tukey post-hoc test were used to determine the effects of treatment as a function of time on body weight and rotarod performance. One-way ANOVA followed by Tukey post-hoc test were used to determine the effect of treatment on muscle weight, muscle fiber diameter, mRNA level and protein level. The effect of treatment on muscle force was determined by using unpaired *t* test. Non-parametric Mann-Whitney test was used to determine the effect of PINTA745 on brain lesions. All values are expressed as means ± SEM. The α-level of significance was set at 0.05 for all comparisons.
